# A Coral- and Goose Down-Inspired Coating with Integrated Anti-Scaling and Heat Retention for Energy Conservation

**DOI:** 10.3390/biomimetics11010022

**Published:** 2026-01-01

**Authors:** Ran Zhao, Zhihao Shang, Xiaosong Deng, Jinze Lan, Jingxin Meng

**Affiliations:** 1 Laboratory of Bio-Inspired Smart Interface Science, Technical Institute of Physics and Chemistry, Chinese Academy of Sciences, Beijing 100190, China; zhaoran22@mails.ucas.ac.cn (R.Z.); dengxiaosong20@mails.ucas.ac.cn (X.D.); 2 University of Chinese Academy of Sciences (UCAS), Beijing 100049, China; 3 Oil & Gas Processing Engineering Department, Sinopec Petroleum Engineering Corporation, Dongying 257026, China; shangzhh1617.osec@sinopec.com; 4 State Key Laboratory of Biopharmaceutical Preparation and Delivery, Institute of Process Engineering, Chinese Academy of Sciences, Beijing 100190, China; lanjinze@ipe.ac.cn

**Keywords:** ion-releasing, anti-scaling, thermal insulation

## Abstract

Scaling and thermal loss on the surfaces of industrial equipment and pipelines usually lead to increased energy consumption and reduced operational efficiency. To solve these severe problems, developing advanced coatings with the dual functions of scale resistance and thermal insulation is an effective approach. Inspired by the antifouling agents released from corals and the thermal insulation of goose down, we herein have developed a bioinspired hollow silica microsphere-based (BHSM) coating, exhibiting the synergistic effect of anti-scaling and thermal insulation properties. The BHSM coating is composed of aluminum phosphate (AP) as an inorganic adhesive and scale inhibitor, and hollow silica microspheres (HSMs) as a thermal insulator. In brief, the effective anti-scaling capability comes from released phosphate ions of AP adhesive for chelating with mineral ions, while the high thermal insulation results from the internal air of the HSMs. Compared to the stainless steel (SS 304), the BHSM coating exhibited ~86% scale reduction. Furthermore, the extremely low thermal conductivity of the HSMs endows the BHSM coating with excellent thermal insulation, resulting in a 20% reduction in heat loss relative to the SS 304 surface. Thus, this work presents a promising strategy for anti-scaling and thermal insulation in industrial equipment and pipelines.

## 1. Introduction

Industrial heat exchange equipment and transmission pipelines are frequently confronted with two major challenges during operation: surface scaling and heat loss [1]. The accumulation of surface scaling not only increases fluid resistance and energy consumption but also leads to severe local corrosion and failure of the equipment [2]. In addition, the direct heat loss from the surfaces of equipment and transmission pipelines results in reduced energy utilization efficiency, thereby causing significant economic losses [3]. Currently, the traditional solution is to address anti-scaling and thermal insulation separately: anti-scaling is achieved through physical and chemical cleaning methods [4], while thermal insulation is realized by installing external thermal insulation layers. However, the existing physical and chemical cleaning methods for descaling have the disadvantages of complex processes and the need for shutdowns, and external thermal insulation layers suffer from drawbacks such as cumbersome installation, large volume, and deteriorated thermal insulation performance after dampening. In recent years, nature-inspired interface materials (e.g., superhydrophobic materials [5,6,7], superhydrophilic materials [8,9,10], and oil-infused materials [11,12,13,14,15,16]) have been extensively studied due to their high efficiency and operational simplicity, but most of them focus solely on anti-scaling and overlook thermal insulation. Therefore, developing a bioinspired interface coating with both anti-scaling and thermal insulation functions is a promising strategy to solve the problems of fouling and energy waste in industrial equipment and pipelines.

Adhesive-based coating techniques have attracted considerable interest owing to their excellent compatibility with diverse substrates and functional components [17,18]. In particular, inorganic phosphate adhesives offer remarkable wear resistance and thermal stability [19,20]. For instance, Guo et al. fabricated a robust superhydrophilic and underwater superoleophobic surface by spraying a mixture of aluminum phosphate (AP) and hydrophilic titanium dioxide nanoparticles onto a stainless steel (SS) mesh, followed by a heat treatment that induced crosslinking between the AP and the metal oxide [21]. Furthermore, Li et al. prepared the same coating on an SS mesh, which maintained its oil–water separation capability even after hundreds of cycles of sandpaper abrasion [22]. In addition, inorganic AP adhesive enables the bonding of oxide and non-oxide ceramics, with the joint remaining stable at temperatures more than 1000 °C [23]. Notably, the cross-linked AP adhesive undergoes slow hydrolysis underwater [24], releasing phosphate groups that have the potential to chelate mineral ions. This characteristic has sparked our interest in its potential for achieving anti-scaling functionality. Typically, incorporating hollow microspheres into coatings can significantly enhance their thermal insulation performance by effectively reducing heat transfer through mechanisms such as blocking heat conduction and reflecting thermal radiation [25,26,27,28]. Therefore, combining AP with hollow microspheres may enable anti-scaling and thermal insulation at the same time.

**Figure 1:**
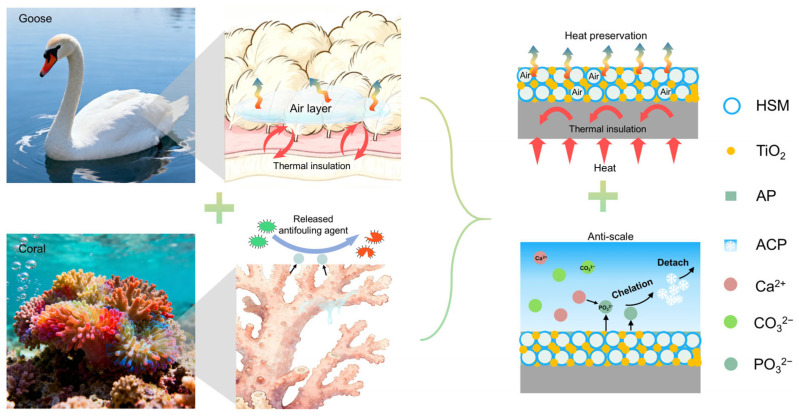
Inspired by the antifouling of corals and the thermal resistance of goose down, a bioinspired dual-functional coating has been developed, exhibiting synergistic anti-scaling and thermal insulation properties.

Inspired by the microbial resistance of the corals [29] and the heat loss blockage of goose down [30], we herein have developed a bioinspired hollow silica microsphere-based (BHSM) coating, exhibiting the synergistic effect of anti-scaling and thermal insulation (Figure 1). This BHSM coating is based on a heat-resistant inorganic AP adhesive and titanium dioxide nanoparticles as the matrix, with HSMs serving as the thermal insulation units. On the one hand, the inorganic AP adhesive can slowly release phosphate ions in water, forming amorphous calcium phosphate (ACP) flocculent precipitates with low adhesion by chelating with mineral ions (e.g., Ca^2+^), thereby achieving effective anti-scaling performance. On the other hand, the dispersed HSMs construct abundant air pockets to realize thermal insulation due to the poor thermal conductivity of air. Compared with the stainless steel (SS 304), the BHSM coating possesses not only excellent anti-scaling performance at a high temperature, but also good thermal insulation performance. Therefore, this study may provide a promising and scalable strategy for solving the dual issues of scaling blockage and heat loss in heat exchange equipment and transportation pipelines.

## 2. Materials and Methods

### 2.1. Fabrication of BHSM

First, AP needs to be freshly synthesized: 85% phosphoric acid (Aladdin, Shanghai, China) is diluted to a concentration of 60%. Subsequently, it is stirred in an oil bath at 100 °C for 3 h, during which aluminum hydroxide (Al(OH)_3_, Macklin, Shanghai, China) is gradually added. The molar ratio of phosphoric acid to Al(OH)_3_ is maintained at 3:1. After 3 h, a colorless and transparent adhesive with aluminum phosphate as the main component is obtained. The aforementioned aluminum phosphate adhesive is uniformly dissolved in deionized water; the dilution parameter in this experiment is as follows: 2 g of aluminum phosphate is dissolved in 5 g of deionized water, and a dilute liquid solution (i.e., AP) is obtained after thorough mixing. Weigh 0.5 g of titanium dioxide nanoparticles (hydrophilic TiO_2_, Aladdin, Shanghai, China) and 0.5 g of HSMs (State Key Laboratory of Technologies in Space Cryogenic Propellants, Beijing, China), mix and disperse them in 15 mL of anhydrous ethanol, then blend the mixture with the dilute liquid solution AP to form a white suspension. The SS 304 substrate is sequentially cleaned by ultrasonication in acetone for 10 min, followed by ultrasonication in ethanol for 10 min, and then rinsed with deionized water, before finally being dried under a nitrogen stream. The suspension is then sprayed onto the surface of clean SS 304 with 0.2 MPa, spray angle of 90° and a distance of ca. 10 cm to obtain coatings. To promote the crosslinking and curing of the coating, the samples are continuously heat-treated at 120 °C for 2 h, followed by another continuous heat treatment at 200 °C for 1 h [21]. Finally, the BHSM is successfully prepared.

### 2.2. Experiment for Scale Deposition

A supersaturated mineral solution of calcium carbonate was prepared by mixing 10 mM calcium chloride (CaCl_2_) and sodium bicarbonate (NaHCO_3_) solutions, followed by stirring for 10 min [31]. A total of 100 mL of the 10 mM supersaturated mineral solution was added to a hydrophobic beaker modified with 1H,1H,2H,2H-perfluorodecyltrimethoxysilane. Subsequently, the samples (1 cm^2^) were placed in the beaker, and the temperature was maintained at 80 °C via a constant temperature water bath. To prevent scaling on the inner walls of the water bath and beakers, as well as to eliminate the interference of total mineral crystal precipitation at high temperatures, the mineral solution was replaced with freshly prepared solution every 12 h. To prevent water loss from the water bath, approximately 1.5 L of deionized water was supplemented every 3 h. The scaled substrate was then removed from the solution, and immersed gently in deionized water to remove any residual mineral solution and unadhered scale. Subsequently, it was dried in a vacuum oven at 50 °C for at least 4 h. The quantitative analysis of the dissolved scaling layers in 20 mM EDTA-Na_2_ dissolution by Inductively Coupled Plasma Atomic Emission Spectroscopy (ICP-AES, Varian 710-OES, Palo Alto, CA, USA). Error bars represent the standard deviation of three independent tests.

### 2.3. Characterization

The surface morphology and element distribution of the coating and scaling were characterized by an Environmental Scanning Electron Microscope (ESEM, Quanta FEG 250, FEI, Hillsboro, OR, USA) and its supporting Energy Dispersive Spectroscopy (EDS, Quanta FEG 250, Hillsboro, OR, USA), respectively. The morphology of calcium carbonate crystals adhered to the surface was further observed using a Metallographic Microscope (KP-RX50M-63M, SOPTOP, Ningbo, China). The quantitative analysis of the phosphate releases and the dissolved scaling layers in 20 mM EDTA-Na_2_ dissolution was performed via Inductively Coupled Plasma Atomic Emission Spectroscopy (ICP-AES, Varian 710-OES, Palo Alto, CA, USA). The wettability of the coating was evaluated by a Contact Angle Measuring Instrument (SCA20, Dataphysics, Filderstadt, Germany). The 3 µL of the liquid was measured for the water contact angle at room temperature using the SCA20 instrument (SCA20, Dataphysics, Filderstadt, Germany). The stable time was defined as 5 s after the liquid droplet state remained unchanged. The error bars represent standard deviation of three individual tests from three samples. The thermal insulation performance was characterized by real-time monitoring of surface temperature changes using an Infrared Thermographic Camera (FLIR A615, Teledyne, Thousand Oaks, CA, USA), the thermal conductivity of the SS 304 was measured using a Laser Flash Apparatus (LFA 467, NETZSCH, NETZSCH-Gerätebau GmbH, Selb, Bavaria, Germany), and the thermal conductivity of HSMs was measured with different diameters by utilizing a Hot Disk Thermal Constant Analyzer (TPS2500S, Hot Disk, Gothenburg, Sweden).

## 3. Results and Discussion

### 3.1. Preparation and Characterization of BHSM Coating

**Figure 2:**
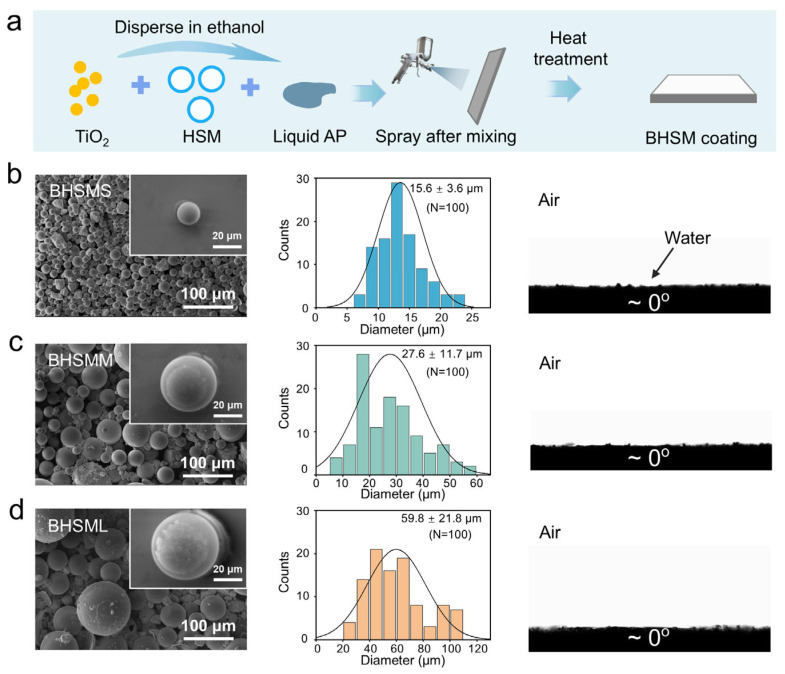
Preparation and characterization of BHSM coatings. (**a**) Schematic of the preparation of the BHSM coating. (**b**–**d**) Surface morphologies, HSM diameters, and surface wettabilities of different BHSM coatings.

To investigate the effect of the diameter of hollow silica microspheres on coating properties such as surface morphology, anti-scaling performance, and thermal insulation performance, three kinds of HSMs with varied diameters (small, medium, and large) were used to fabricate three types of BHSM coatings including BHSMS, BHSMM, and BHSML. As shown in Figure 2a, the ethanol/water mixed solution containing inorganic AP adhesive, hydrophilic TiO_2_ nanoparticles, and HSMs was first spray-coated onto SS 304 substrates, and then the BHSM coatings in Figure S1 were obtained through subsequent heat treatment. Environmental scanning electron microscopy (ESEM) images show the surface morphology of the three BHSM coatings prepared with three types of HSMs (Figure 2b–d). A significantly denser structure was observed in BHSMS, which was prepared with the smaller-diameter HSMs. The diameters of the HSMs were determined by statistically analyzing 100 particles via Image J 2.0 software. The average diameters for the small-, medium-, and large-size HSM were approximately 15.6, 27.6, and 59.8 μm, respectively. Furthermore, ESEM images of fractured HSMs reveal their internal hollow structure (Figure S2). The water contact angles of three HSMs with different diameters was 61.5 ± 2.9° for HSMS, 63.1 ± 3.0° for HSMM, 61.3 ± 1.4° for HSML, respectively (Figure S3). After preparing the BHSM coating, all three as-prepared BHSM coatings show similar surface wettability with the water contact angles of ~0°.

Durability refers to the coating’s ability to withstand external factors such as mechanical actions. The durability of the coating was evaluated using peel-off of tape (ASTM D903) and sandpaper abrasion [32,33]. Accordingly, a tape peeling test using 3M tape was conducted to evaluate the interfacial adhesion strength of the coating. As shown in Figure S4a, the adhesion strength of the no-heat-treatment coating was only 0.28 ± 0.12 N, while the BHSM coatings were more than 9.40 ± 0.31 N for BHSMS, 8.92 ± 0.67 N for BHSMM, and 7.29 ± 1.19 N for BHSML, respectively. Furthermore, it can be observed that the surface of the BHSMS coating was not undamaged after the test (Figure S4b). To access the wear resistance [33], we conducted a sandpaper abrasion test from 10 to 50 cycles by using 1000-mesh sandpaper under the pressure of a 100 g weight. As shown in Figure S5, the optical images of BHSM (e.g., BHSMS) coating exhibit little damage after sandpaper abrasion. To demonstrate the applicability of the coating to multiple substrates, BHSMS coatings were successfully prepared on other substrate such as carbon steel (CS), copper (Cu), and aluminum (Al), as shown in Figure S6. In addition, a tape peeling test using 3M tape was conducted to evaluate the interfacial adhesion strength of the coating. As shown in Figure S7, the BHSM (e.g., BHSMS) coatings were more than 8.38 ± 0.35 N on CS, 7.80 ± 0.23 N on Cu, and 6.24 ± 0.55 N on Al, respectively. These results indicate that the coating exhibits excellent adhesion on various metallic substrates, demonstrating good substrate compatibility. Therefore, three robust coatings with HSMs of different diameters were successfully fabricated for subsequent investigation of their anti-scaling and thermal insulation performance.

### 3.2. Anti-Scale Mechanism and Performance of BHSM

**Figure 3:**
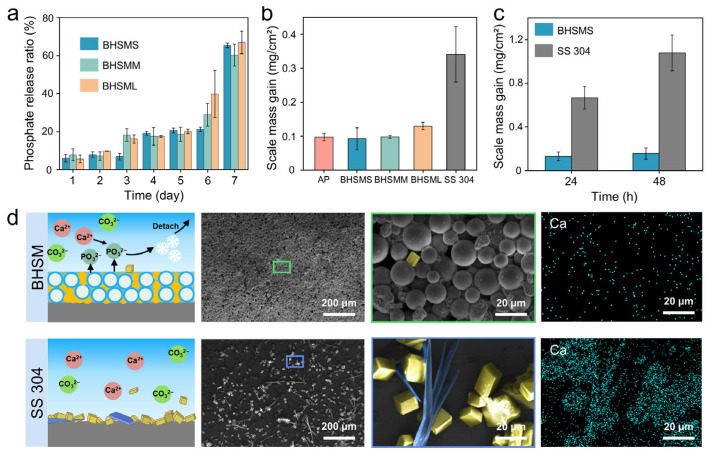
Anti-scale performance of BHSM coatings. (**a**) Phosphate release ratio of three types of BHSM coatings over time. (**b**) Scale resistance of three BHSM coatings, AP and bare SS 304 after 12 h scaling test. (**c**) Comparison of anti-scaling performance between BHSMS and SS 304. (**d**) Schematic, morphology, and corresponding EDS mappings of the BHSMS and SS 304 after scaling test at 80 °C for 12 h.

To illustrate the role of the inorganic AP adhesive in scale inhibition—a mechanism whereby it slowly releases phosphate ions that chelate calcium ions in the mineral solution to form low-adhesion calcium phosphate—we first measured the phosphate release ratio from the three types of coatings (i.e., BHSMS, BHSMM, and BHSML) in water over time from 1 to 7 days. The quantitative analysis of phosphate release was carried out using Inductively Coupled Plasma Atomic Emission Spectrometry (ICP-AES). As shown in Figure 3a, the phosphate release rates of the three coatings increased from 6.06 ± 1.98%, 7.80 ± 3.16%, and 5.67 ± 2.02% on Day 1 to 65.40 ± 1.23%, 60.29 ± 5.77%, and 66.96 ± 5.92% on Day 7, respectively. Consequently, there were no significant difference among the phosphate release ratios of these three coatings from the inorganic AP adhesive. Moreover, we compared the anti-scaling properties of the three coatings and SS 304 after scaling in an 80 °C supersaturated calcium carbonate (CaCO_3_) solution for 12 h. The quantitative analysis of the dissolved scaling layers was performed via ICP-AES. As shown in Figure 3b, ICP-AES results show that the scaling mass gain (SMG) values were 0.10 ± 0.01 mg/cm^2^ for pure AP, 0.09 ± 0.03 mg/cm^2^ for BHSMS, 0.10 ± 0.004 mg/cm^2^ for BHSMM, and 0.13 ± 0.01 mg/cm^2^ for BHSML, respectively. All coatings demonstrated significantly lower SMG compared to the bare SS 304 (0.34 ± 0.08 mg/cm^2^), showing their excellent anti-scaling performance. The pure AP coating demonstrated anti-scaling performance similar to that of the three as-prepared BHSM coatings. These results suggest that the superior anti-scaling performance of the coatings is primarily attributed to the release of phosphate ions. In addition, ESEM and EDS images in Figure 3d reveal minimal scale deposition on the coating after the 12 h scaling test. In contrast, the SS 304 surface was extensively covered by a mixture of block-shaped (yellow) and needle-shaped (blue) scale crystals. Furthermore, a 12 h mineral deposition experiment was conducted on the BHSM (e.g., BHSMS) coating at different temperatures (20 °C, 40 °C, and 60 °C) to demonstrate its anti-scaling performance. As shown in Figure S8, the SMG of the BHSM coating was 0.073 ± 0.009 mg/cm^2^ at 20 °C, 0.070 ± 0.011 mg/cm^2^ at 40 °C, and 0.091 ± 0.043 mg/cm^2^ at 60 °C, respectively. The BHSM coating demonstrated effective anti-scaling performance at various temperatures. To evaluate the anti-scaling performance with varied times, the BHSM coating was subjected to an extended 48 h scaling test by replacing freshly prepared mineral solution every 12 h (Figure 3c). It exhibited negligible scale accumulation (0.16 ± 0.05 mg/cm^2^), which was drastically lower than the massive deposition on the SS 304 substrate (1.08 ± 0.16 mg/cm^2^). Meanwhile, only trace amounts of scale accumulation were observed on the BHSM, while the SS 304 surface was covered with a large amount of scale (color) in Figure S9. Furthermore, a continuous 48 h mineral deposition experiment was conducted without replacing the mineral solution (Figure S10). The SMG on the BHSMS coating and the SS 304 substrate were 0.12 ± 0.02 mg/cm^2^ and 0.99 ± 0.14 mg/cm^2^, respectively. The coating demonstrated sustained anti-scaling performance during the 48 h continuous experiment compared to the SS 304. Therefore, the BHSM coating maintains excellent anti-scaling performance under long-term high-temperature conditions.

### 3.3. Thermal Insulation of BHSM

**Figure 4:**
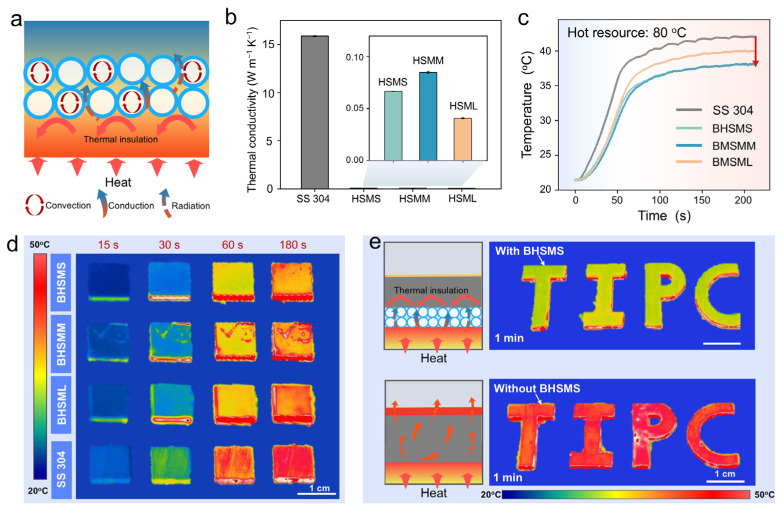
Thermal insulation of BHSM coatings. (**a**) Schematic of thermal insulation mechanism of the BHSM. (**b**) Thermal conductivity of three types of HSMs with different diameters (e.g., HSMS, HSMM, and HSML). (**c**,**d**) Thermal insulation results of the BHSM coatings and SS 304 from an Infrared Thermographic Camera. (**e**) IR thermograms of “TIPC” letters on SS 304 substrate with and without BHSMS coatings (the diameter of 15.6 ± 3.6 μm).

The thermal insulation function is crucial for the heat preservation of the heat exchange equipment and pipeline transportation. As shown in Figure 4a, the composite structure co-constructed by the heat-resisting AP adhesive and HSMs may endow the BHSM coating with excellent thermal insulation performance. The AP adhesive forms a continuous and robust three-dimensional network framework, which uniformly encapsulates and immobilizes a large number of HSMs within it. These structures efficiently inhibit heat transfer through multiple synergistic mechanisms: the AP adhesive framework itself constructs tortuous and extended heat flow paths, significantly retarding solid thermal conduction; meanwhile, the HSMs act as isolated air cavity units that seal static air inside, greatly reducing the overall thermal conductivity of the material. Furthermore, dense interfacial regions are formed between the microspheres and the adhesive matrix, creating additional thermal resistance points. In terms of thermal radiation shielding, the HSMs effectively scatter and reflect infrared radiation by virtue of their spherical geometry and shell characteristics, and their uniform distribution further enhances the suppression of radiative heat transfer in three dimensions. Meanwhile, the combined effect of the continuous network formed by the heat-resisting AP adhesive and the closed air pockets divided by microsphere stacking greatly restricts the convective motion of air inside the material, thereby limiting convective heat transfer. This synergistic barrier effect integrating the three heat transfer forms (conduction, radiation, and convection) may endow the material with comprehensive and efficient multi-level thermal barrier properties [30,34]. Figure 4b shows the thermal conductivity of HSMs with different diameters by utilizing a Hot Disk Thermal Constant Analyzer (TPS2500S, Hot Disk, Gothenburg, Sweden), and the thermal conductivity of the SS 304 substrate was measured using a Laser Flash Apparatus (LFA 467, NETZSCH, NETZSCH-Gerätebau GmbH, Bavaria, Germany). The thermal conductivity of HSMs with diameters varying from small (HSMS, ~0.07 W m^−1^ K^−1^), to medium (HSMM, ~0.04 W m^−1^ K^−1^), to large (HSML, ~0.08 W m^−1^ K^−1^) is much lower than that of the bare SS 304 (15.87 ± 0.04 W m^−1^ K^−1^). Meanwhile, the thermal conductivities of the three types of microspheres are very close to that of air (~0.03 W m^−1^ K^−1^). The extremely low thermal conductivity of the HSMs may confer excellent thermal insulation properties onto the BHSM coating.

To intuitively evaluate the thermal insulation performance, the heat transfer process of the coating on a hot stage was observed in real time using an Infrared Thermographic Camera (FLIR A615, Teledyne, Thousand Oaks, CA, USA). In this test, the thickness of the three selected coatings was approximately 150 μm. As shown in Figure S11, the thicknesses of BHSM coatings were 154.2 ± 4.2 μm for BHSMS, 148.1 ± 4.9 μm for BHSML, and 153.6 ± 10.4 μm for BHSML, respectively. The temperature–time curves in Figure 4c reveal the difference in the thermal insulation among the coatings and the SS 304. All coatings exhibited superior thermal insulation performance, showing significantly lower heating rates, and their top surface temperatures were consistently lower than that of the SS 304. Furthermore, the surface temperatures of BHSMS and BHSMM are lower than that of BHSML, which may be due to the fact that the coatings prepared with HSMs of smaller diameters are denser. The infrared thermograms in Figure 4d clearly show the macroscopic temperature rise process of the SS 304 surface and the three-coatings surface. During the entire heating stage, the temperature rise rate of the SS 304 surface was significantly higher than that of all coatings. Specifically, after 30 s heating, the SS 304 surface had turned light yellow (indicating a relatively high temperature), while the surfaces of the three coatings still remained blue; by 60 s, the SS 304 surface had further changed to orange-red, and the coating surfaces were only dark yellow; at the end of heating, the SS 304 surface had reached deep red, while the highest temperature on the coating surfaces was only shown as orange-yellow. Figure 4e demonstrates the practical application of the BHSM coating. A specially customized TIPC built from the BHSMS coating with HSMs represent abbreviation of “Technical Institute of Physics and Chemistry” were coated on SS 304 sheet. After placing on an 80 °C hot plate for 1 min, the BHSMS surface was observed to be orange-yellow, exhibiting a lower temperature than the pure red SS 304 surface. These results clearly indicate that the BHSM coating can effectively retard temperature rise and exhibit significant thermal insulation.

## 4. Conclusions

In summary, we have developed a BHSM coating with excellent anti-scaling and thermal insulation capabilities. It is fabricated using heat-resistant inorganic AP adhesive to consolidate thermally insulating hollow HSMs. The adhesive serves a dual purpose: it imparts excellent durability by bonding the structure and inhibits scale deposition by releasing phosphate ions that chelate calcium ions. The extremely low thermal conductivity of the HSM endows the BHSM coating with excellent thermal insulation performance, thereby resulting in ca. 20% reduction in heat loss. Therefore, this study offers a promising and scalable strategy for addressing the dual issues of scaling-induced blockage and heat loss in heat exchange equipment and transportation pipelines.

## Data Availability

Data are contained within the article and Supplementary Materials.

## Supplementary Materials

The following supporting information can be downloaded at: https://www.mdpi.com/article/10.3390/biomimetics11010022/s1, Figure S1: Optical images of SS 304 and three as-prepared BHSM coatings including BHSMS, BHSMM, and BHSML. Figure S2: ESEM images of HSMs with internal hollow structure. Figure S3: Surface wettability of HSMs with different sizes. Figure S4: The tape peeling test for evaluating the interfacial adhesion strength of the BHSM coating. Figure S5: The abrasion resistance test of BHSM coating. Figure S6: The preparation of the BHSM (e.g. BHSMS) coating on different substrates. Figure S7: The interfacial adhesion of BHSM (e.g., BHSMS) coating on different substrates. Figure S8: The anti-scaling performance of BHSMS coating at different temperatures after 12 h scaling test. Figure S9: Cross-polarized light micrograph of BHSM and SS after scaling test from 12 to 48 hours. Figure S10: The anti-scaling performance of the BHSMS coating was investigated in a 48-hour continuous mineral deposition experiment. Figure S11: The three types of as-prepared BHSM coatings with similar thickness.

## Abbreviations

The following abbreviations are used in this manuscript:

BHSM	Bioinspired hollow silica microsphere-based
HSM	Hollow silica microsphere
AP	Aluminum phosphate
TiO_2_	Titanium dioxide
ACP	Amorphous calcium phosphate
SS 304	Stainless steel 304
