# Supplementary material for: A Coral- and Goose Down-Inspired Coating with Integrated Anti-Scaling and Heat Retention for Energy Conservation
Source: Biomimetics (Basel). 2026 Jan 1;11(1):22. doi: 10.3390/biomimetics11010022 (PMC12839186; doi:10.3390/biomimetics11010022)
Supplement: Supplementary file 1 [file biomimetics-11-00022-s001.zip › biomimetics-4025651-supplementary.pdf]

# A Coral- and Goose Down-inspired Coating with Integrated Anti-scaling and Heat Retention for Energy Conservation

Ran Zhao[1,4,†], Zhihao Shang[2,†], Xiaosong Deng[1,4,†], Jinze Lan[3] and Jingxin Meng[1,4,*]

[1]Laboratory of Bio-inspired Smart Interface Science, Technical Institute of Physics and Chemistry, Chinese Academy of Sciences, Beijing, 100190, P. R. China.

[2]Oil & Gas Processing Engineering Department, Sinopec Petroleum Engineering Corporation, Dongying, 257026, P. R. China.

[3]State Key Laboratory of Biopharmaceutical Preparation and Delivery, institute of Process Engineering, Chinese Academy of Sciences, Beijing, 100190, P. R. China

[4]University of Chinese Academy of Sciences (UCAS), Beijing, 100049, P. R. China

[*]Correspondence: Jingxin Meng, E-mail: mengjx628@mail.ipc.ac.cn

[†]These authors contributed equally to this work.

**Supplementary Materials**

**Supplementary Note**

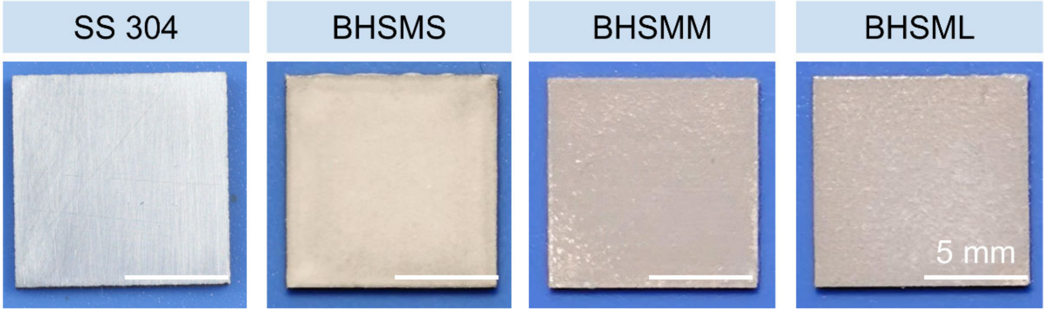


**Figure S1.** Optical images of SS 304 and three as-prepared BHSM coatings including BHSMS, BHSMM, and BHSML.

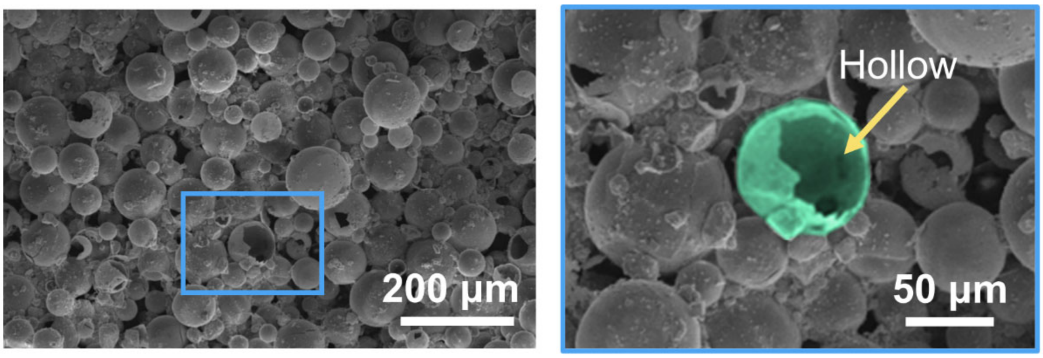


**Figure S2:** ESEM images of HSMs with internal hollow structure. The pseudo-color part shows a damaged microsphere, demonstrating that its interior is hollow.

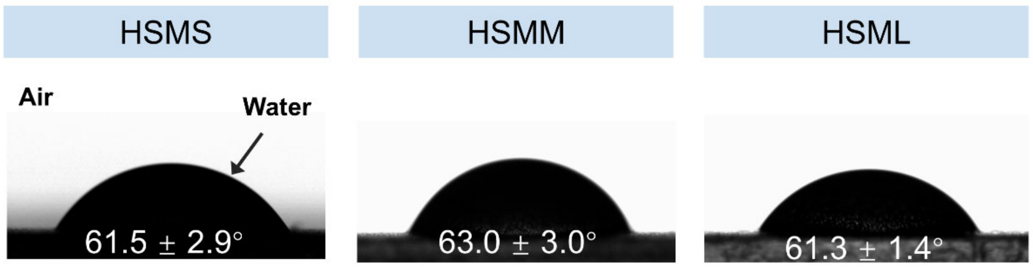


**Figure S3.** Surface wettability of HSMs with different sizes.

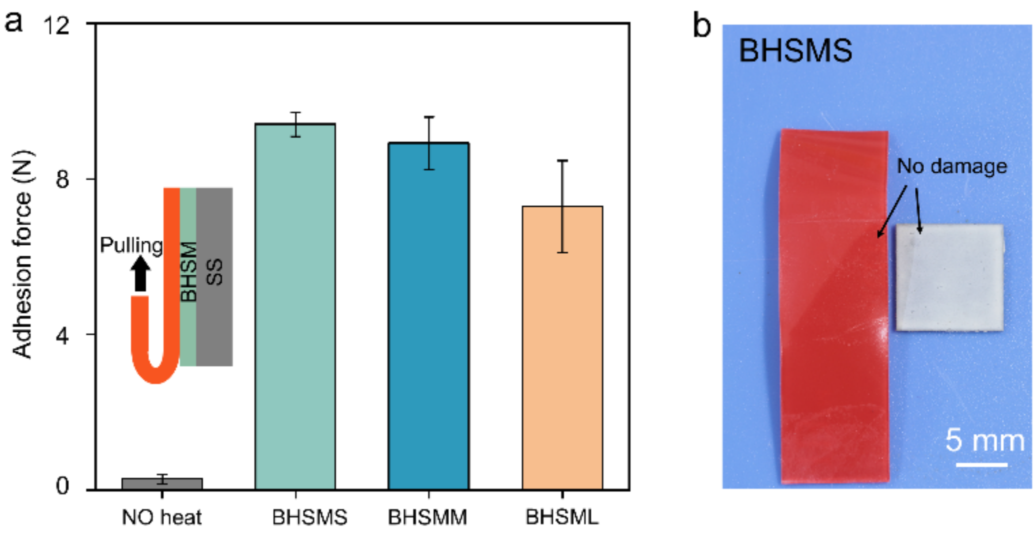


**Figure S4.** The tape peeling test for evaluating the interfacial adhesion strength of the BHSM coating. (a) The adhesion force of BHSM coating. (b) The optical image of BHSMS coating after tape-peeling test.

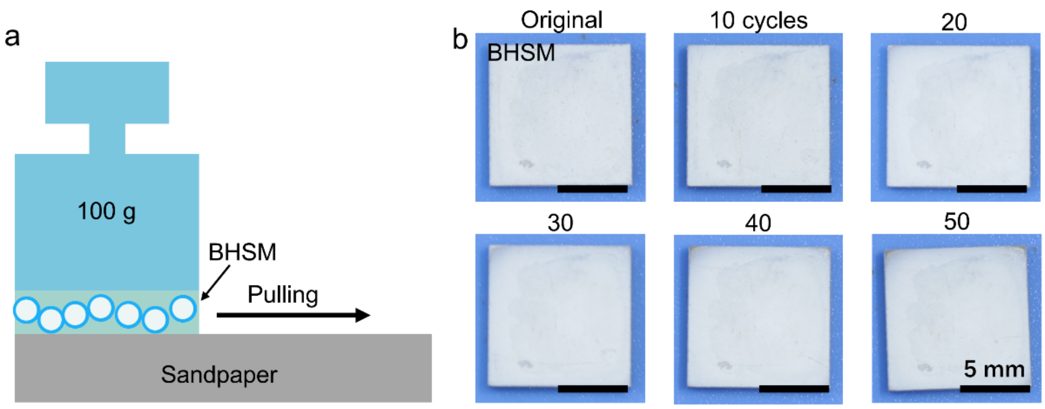


**Figure S5.** The abrasion resistance test of BHSM coating. The schematic diagram (a) and the optical image (b) of the abrasion test of BHSM (e.g., BHSMS).

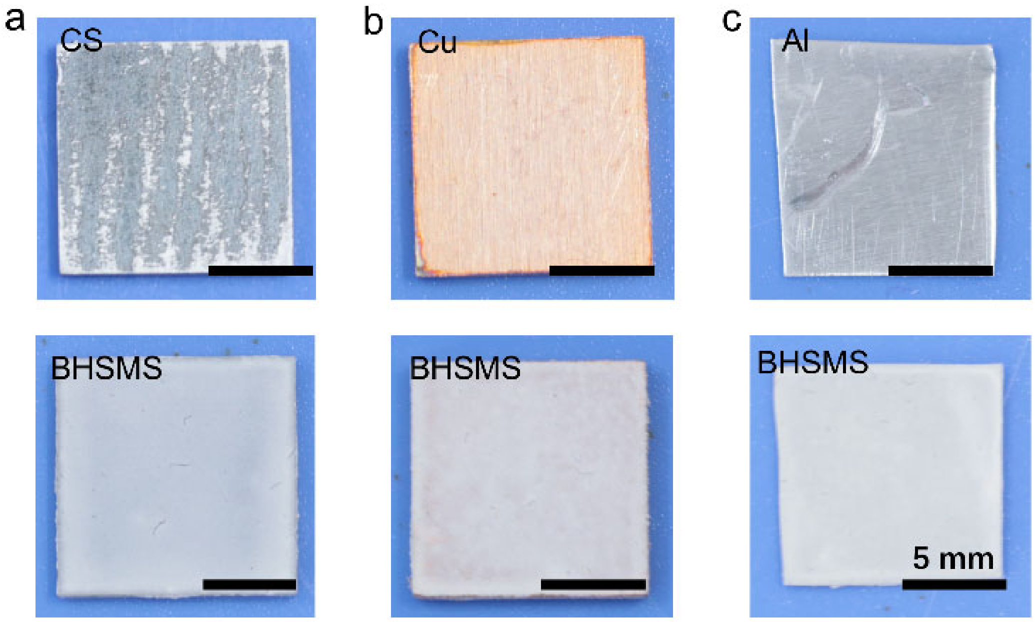


**Figure S6.** The preparation of the BHSM (e.g. BHSMS) coating on different substrates of (a) CS, (b) Cu and (c) Al.

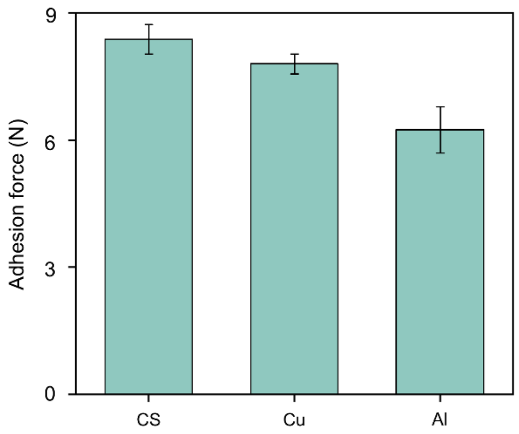


**Figure S7.** The interfacial adhesion of BHSM (e.g., BHSMS) coating on different substrates.

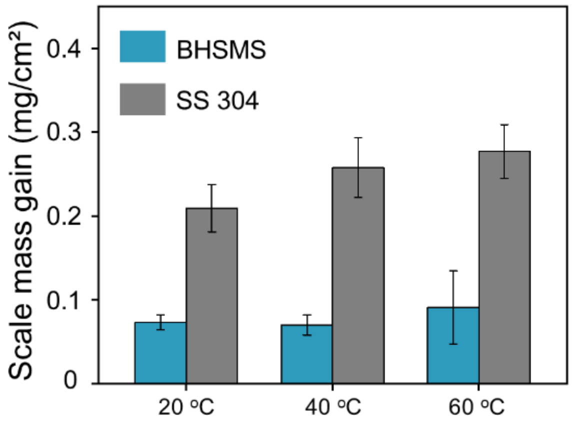


**Figure S8.** The anti-scaling performance of BHSMS coating and SS 304 at different temperatures after 12 h scaling test.

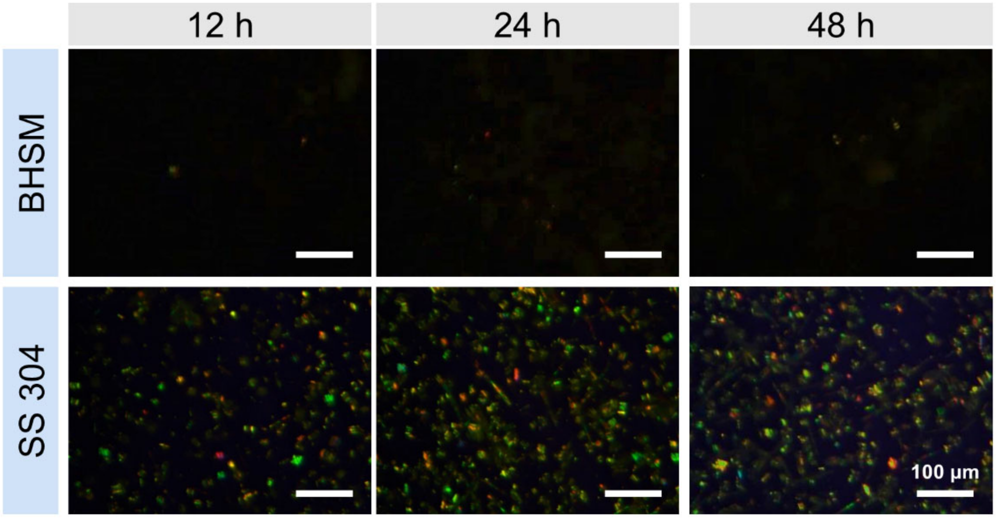


**Figure S9.** Cross-polarized light micrograph of BHSM and SS after scaling test from 12 to 48 h.

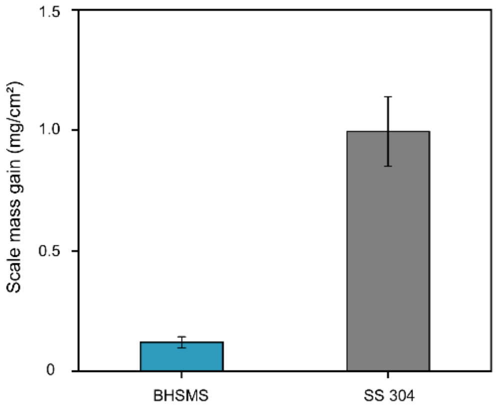


**Figure S10.** The anti-scaling performance of the BHSMS coating was investigated in a 48-hour continuous mineral deposition experiment.

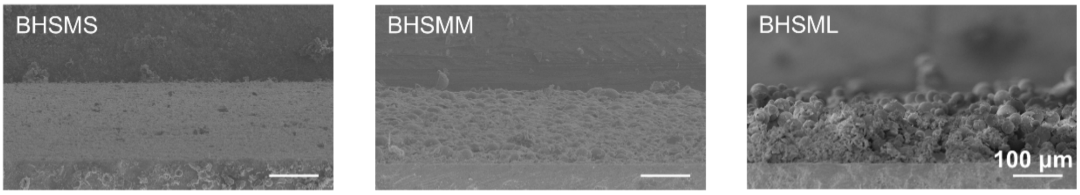


**Figure S11.** The three types of as-prepared BHSM coatings with similar thickness.

**Supplementary Note**

The ~20% reduction in heat loss was calculated based on the final surface temperature of the coating shown in Figure 4c.

$$(\Delta T_{SS} - \Delta T_{BHSMS})/\, \Delta T_{SS} \times 100\% = \eta \qquad \text{(E1)}$$

where $\Delta T_{SS}$ represents the temperature difference between SS 304 after heating and its initial temperature, $\Delta T_{BHSMS}$ represents the temperature difference between BHSMS after heating and its initial temperature, $\eta$ represents the heat loss ratio.

Take heating after 200 seconds as an example, $\Delta T_{SS}$ = 42.00 °C (temperature after heating) - 21.51 °C (initial temperature), $\Delta T_{BHSMS}$ = 38.03 °C (temperature after heating) – 21.51 °C (initial temperature). According to E1, $\eta$ value yields a result of 19.4% (~20%).
